# Supplementary material for: Pyruvate Dehydrogenase Kinase 1 inhibition mediated oxidative phosphorylation enhancement in cartilage promotes osteoarthritis progression
Source: BMC Musculoskelet Disord. 2023 Jul 20;24:597. doi: 10.1186/s12891-023-06585-6 (PMC10357736; doi:10.1186/s12891-023-06585-6)
Supplement: Supplementary file 1 — Additional file 1. [file 12891_2023_6585_MOESM1_ESM.zip › KEGG.Gsea.1653622432464/KEGG_ADHERENS_JUNCTION.html]

Details for gene set KEGG\_ADHERENS\_JUNCTION[GSEA]

|  || Dataset | 55457\_collapsed\_to\_symbols.cls.cls #OA\_versus\_normal.cls.cls #OA\_versus\_normal\_repos |
| Phenotype | cls.cls#OA\_versus\_normal\_repos |
| Upregulated in class | normal |
| GeneSet | KEGG\_ADHERENS\_JUNCTION |
| Enrichment Score (ES) | -0.42509425 |
| Normalized Enrichment Score (NES) | -1.7430617 |
| Nominal p-value | 0.0 |
| FDR q-value | 0.048774764 |
| FWER p-Value | 0.231 |
Table: GSEA Results Summary

  

Fig 1: Enrichment plot: KEGG\_ADHERENS\_JUNCTION      
 Profile of the Running ES Score & Positions of GeneSet Members on the Rank Ordered List

  

| SYMBOL | TITLE | RANK IN GENE LIST | RANK METRIC SCORE | RUNNING ES | CORE ENRICHMENT || 1 | SMAD2 | SMAD family member 2 [Source:HGNC Symbol;Acc:HGNC:6768] | 275 | 0.768 | 0.0089 | No |
| 2 | MAPK3 | mitogen-activated protein kinase 3 [Source:HGNC Symbol;Acc:HGNC:6877] | 757 | 0.579 | -0.0057 | No |
| 3 | ERBB2 | erb-b2 receptor tyrosine kinase 2 [Source:HGNC Symbol;Acc:HGNC:3430] | 963 | 0.526 | -0.0009 | No |
| 4 | MAP3K7 | mitogen-activated protein kinase kinase kinase 7 [Source:HGNC Symbol;Acc:HGNC:6859] | 1701 | 0.399 | -0.0426 | No |
| 5 | CSNK2B | casein kinase 2 beta [Source:HGNC Symbol;Acc:HGNC:2460] | 1788 | 0.385 | -0.0341 | No |
| 6 | CTNNA3 | catenin alpha 3 [Source:HGNC Symbol;Acc:HGNC:2511] | 2030 | 0.358 | -0.0387 | No |
| 7 | ACTN2 | actinin alpha 2 [Source:HGNC Symbol;Acc:HGNC:164] | 2532 | 0.299 | -0.0660 | No |
| 8 | CDH1 | cadherin 1 [Source:HGNC Symbol;Acc:HGNC:1748] | 2589 | 0.292 | -0.0588 | No |
| 9 | WASF2 | WASP family member 2 [Source:HGNC Symbol;Acc:HGNC:12733] | 3101 | 0.239 | -0.0892 | No |
| 10 | PTPN6 | protein tyrosine phosphatase non-receptor type 6 [Source:HGNC Symbol;Acc:HGNC:9658] | 3434 | 0.208 | -0.1068 | No |
| 11 | RAC3 | Rac family small GTPase 3 [Source:HGNC Symbol;Acc:HGNC:9803] | 3849 | 0.171 | -0.1324 | No |
| 12 | CTNNA1 | catenin alpha 1 [Source:HGNC Symbol;Acc:HGNC:2509] | 4003 | 0.156 | -0.1381 | No |
| 13 | SMAD3 | SMAD family member 3 [Source:HGNC Symbol;Acc:HGNC:6769] | 4061 | 0.150 | -0.1367 | No |
| 14 | CTNND1 | catenin delta 1 [Source:HGNC Symbol;Acc:HGNC:2515] | 4243 | 0.133 | -0.1455 | No |
| 15 | ACP1 | acid phosphatase 1 [Source:HGNC Symbol;Acc:HGNC:122] | 4772 | 0.089 | -0.1831 | No |
| 16 | PTPRF | protein tyrosine phosphatase receptor type F [Source:HGNC Symbol;Acc:HGNC:9670] | 4839 | 0.084 | -0.1850 | No |
| 17 | TCF7L2 | transcription factor 7 like 2 [Source:HGNC Symbol;Acc:HGNC:11641] | 5034 | 0.070 | -0.1973 | No |
| 18 | TGFBR1 | transforming growth factor beta receptor 1 [Source:HGNC Symbol;Acc:HGNC:11772] | 5280 | 0.050 | -0.2145 | No |
| 19 | RAC2 | Rac family small GTPase 2 [Source:HGNC Symbol;Acc:HGNC:9802] | 6098 | -0.013 | -0.2777 | No |
| 20 | CSNK2A2 | casein kinase 2 alpha 2 [Source:HGNC Symbol;Acc:HGNC:2459] | 6105 | -0.013 | -0.2776 | No |
| 21 | WAS | WASP actin nucleation promoting factor [Source:HGNC Symbol;Acc:HGNC:12731] | 6666 | -0.054 | -0.3191 | No |
| 22 | NECTIN3 | nectin cell adhesion molecule 3 [Source:HGNC Symbol;Acc:HGNC:17664] | 6767 | -0.061 | -0.3245 | No |
| 23 | MAPK1 | mitogen-activated protein kinase 1 [Source:HGNC Symbol;Acc:HGNC:6871] | 6853 | -0.068 | -0.3285 | No |
| 24 | SRC | "SRC proto-oncogene, non-receptor tyrosine kinase [Source:HGNC Symbol;Acc:HGNC:11283]" | 6940 | -0.075 | -0.3322 | No |
| 25 | AFDN | "afadin, adherens junction formation factor [Source:HGNC Symbol;Acc:HGNC:7137]" | 7077 | -0.086 | -0.3394 | No |
| 26 | RAC1 | Rac family small GTPase 1 [Source:HGNC Symbol;Acc:HGNC:9801] | 7192 | -0.096 | -0.3446 | No |
| 27 | MET | "MET proto-oncogene, receptor tyrosine kinase [Source:HGNC Symbol;Acc:HGNC:7029]" | 7322 | -0.104 | -0.3505 | No |
| 28 | NLK | nemo like kinase [Source:HGNC Symbol;Acc:HGNC:29858] | 7336 | -0.105 | -0.3474 | No |
| 29 | TCF7L1 | transcription factor 7 like 1 [Source:HGNC Symbol;Acc:HGNC:11640] | 7383 | -0.109 | -0.3466 | No |
| 30 | LEF1 | lymphoid enhancer binding factor 1 [Source:HGNC Symbol;Acc:HGNC:6551] | 7467 | -0.116 | -0.3485 | No |
| 31 | ACTN3 | actinin alpha 3 [Source:HGNC Symbol;Acc:HGNC:165] | 7557 | -0.124 | -0.3506 | No |
| 32 | VCL | vinculin [Source:HGNC Symbol;Acc:HGNC:12665] | 7687 | -0.133 | -0.3554 | No |
| 33 | FGFR1 | fibroblast growth factor receptor 1 [Source:HGNC Symbol;Acc:HGNC:3688] | 7905 | -0.148 | -0.3665 | No |
| 34 | PTPRB | protein tyrosine phosphatase receptor type B [Source:HGNC Symbol;Acc:HGNC:9665] | 8044 | -0.158 | -0.3710 | No |
| 35 | TGFBR2 | transforming growth factor beta receptor 2 [Source:HGNC Symbol;Acc:HGNC:11773] | 8241 | -0.174 | -0.3794 | No |
| 36 | IQGAP1 | IQ motif containing GTPase activating protein 1 [Source:HGNC Symbol;Acc:HGNC:6110] | 8276 | -0.177 | -0.3750 | No |
| 37 | ACTN1 | actinin alpha 1 [Source:HGNC Symbol;Acc:HGNC:163] | 8385 | -0.184 | -0.3762 | No |
| 38 | SSX2IP | SSX family member 2 interacting protein [Source:HGNC Symbol;Acc:HGNC:16509] | 8489 | -0.193 | -0.3766 | No |
| 39 | SORBS1 | sorbin and SH3 domain containing 1 [Source:HGNC Symbol;Acc:HGNC:14565] | 8509 | -0.194 | -0.3704 | No |
| 40 | TJP1 | tight junction protein 1 [Source:HGNC Symbol;Acc:HGNC:11827] | 8523 | -0.195 | -0.3637 | No |
| 41 | RHOA | ras homolog family member A [Source:HGNC Symbol;Acc:HGNC:667] | 8601 | -0.201 | -0.3617 | No |
| 42 | PTPN1 | protein tyrosine phosphatase non-receptor type 1 [Source:HGNC Symbol;Acc:HGNC:9642] | 8829 | -0.220 | -0.3707 | No |
| 43 | SMAD4 | SMAD family member 4 [Source:HGNC Symbol;Acc:HGNC:6770] | 9035 | -0.235 | -0.3774 | No |
| 44 | PTPRJ | protein tyrosine phosphatase receptor type J [Source:HGNC Symbol;Acc:HGNC:9673] | 9463 | -0.270 | -0.4001 | No |
| 45 | SNAI2 | snail family transcriptional repressor 2 [Source:HGNC Symbol;Acc:HGNC:11094] | 9697 | -0.291 | -0.4067 | No |
| 46 | BAIAP2 | BAR/IMD domain containing adaptor protein 2 [Source:HGNC Symbol;Acc:HGNC:947] | 9892 | -0.311 | -0.4096 | No |
| 47 | FYN | "FYN proto-oncogene, Src family tyrosine kinase [Source:HGNC Symbol;Acc:HGNC:4037]" | 10092 | -0.330 | -0.4120 | Yes |
| 48 | LMO7 | LIM domain 7 [Source:HGNC Symbol;Acc:HGNC:6646] | 10108 | -0.331 | -0.4001 | Yes |
| 49 | NECTIN2 | nectin cell adhesion molecule 2 [Source:HGNC Symbol;Acc:HGNC:9707] | 10204 | -0.341 | -0.3941 | Yes |
| 50 | WASF3 | WASP family member 3 [Source:HGNC Symbol;Acc:HGNC:12734] | 10316 | -0.352 | -0.3888 | Yes |
| 51 | ACTN4 | actinin alpha 4 [Source:HGNC Symbol;Acc:HGNC:166] | 10472 | -0.370 | -0.3863 | Yes |
| 52 | NECTIN1 | nectin cell adhesion molecule 1 [Source:HGNC Symbol;Acc:HGNC:9706] | 10771 | -0.402 | -0.3936 | Yes |
| 53 | CREBBP | CREB binding protein [Source:HGNC Symbol;Acc:HGNC:2348] | 10881 | -0.414 | -0.3858 | Yes |
| 54 | PTPRM | protein tyrosine phosphatase receptor type M [Source:HGNC Symbol;Acc:HGNC:9675] | 11289 | -0.470 | -0.3989 | Yes |
| 55 | CTNNA2 | catenin alpha 2 [Source:HGNC Symbol;Acc:HGNC:2510] | 11342 | -0.477 | -0.3841 | Yes |
| 56 | WASF1 | WASP family member 1 [Source:HGNC Symbol;Acc:HGNC:12732] | 11387 | -0.484 | -0.3684 | Yes |
| 57 | EP300 | E1A binding protein p300 [Source:HGNC Symbol;Acc:HGNC:3373] | 11741 | -0.540 | -0.3746 | Yes |
| 58 | CSNK2A1 | casein kinase 2 alpha 1 [Source:HGNC Symbol;Acc:HGNC:2457] | 11895 | -0.570 | -0.3641 | Yes |
| 59 | FER | FER tyrosine kinase [Source:HGNC Symbol;Acc:HGNC:3655] | 12101 | -0.620 | -0.3555 | Yes |
| 60 | EGFR | epidermal growth factor receptor [Source:HGNC Symbol;Acc:HGNC:3236] | 12109 | -0.622 | -0.3315 | Yes |
| 61 | WASL | WASP like actin nucleation promoting factor [Source:HGNC Symbol;Acc:HGNC:12735] | 12207 | -0.647 | -0.3135 | Yes |
| 62 | FARP2 | "FERM, ARH/RhoGEF and pleckstrin domain protein 2 [Source:HGNC Symbol;Acc:HGNC:16460]" | 12318 | -0.682 | -0.2951 | Yes |
| 63 | SNAI1 | snail family transcriptional repressor 1 [Source:HGNC Symbol;Acc:HGNC:11128] | 12365 | -0.696 | -0.2712 | Yes |
| 64 | YES1 | "YES proto-oncogene 1, Src family tyrosine kinase [Source:HGNC Symbol;Acc:HGNC:12841]" | 12450 | -0.725 | -0.2491 | Yes |
| 65 | PARD3 | par-3 family cell polarity regulator [Source:HGNC Symbol;Acc:HGNC:16051] | 12489 | -0.743 | -0.2227 | Yes |
| 66 | ACTG1 | actin gamma 1 [Source:HGNC Symbol;Acc:HGNC:144] | 12613 | -0.806 | -0.2005 | Yes |
| 67 | TCF7 | transcription factor 7 [Source:HGNC Symbol;Acc:HGNC:11639] | 12628 | -0.813 | -0.1695 | Yes |
| 68 | CDC42 | cell division cycle 42 [Source:HGNC Symbol;Acc:HGNC:1736] | 12681 | -0.852 | -0.1399 | Yes |
| 69 | IGF1R | insulin like growth factor 1 receptor [Source:HGNC Symbol;Acc:HGNC:5465] | 12689 | -0.855 | -0.1067 | Yes |
| 70 | ACTB | actin beta [Source:HGNC Symbol;Acc:HGNC:132] | 12757 | -0.920 | -0.0755 | Yes |
| 71 | INSR | insulin receptor [Source:HGNC Symbol;Acc:HGNC:6091] | 12856 | -1.071 | -0.0409 | Yes |
| 72 | CTNNB1 | catenin beta 1 [Source:HGNC Symbol;Acc:HGNC:2514] | 12866 | -1.119 | 0.0027 | Yes |
Table: GSEA details [plain text format]

  

Fig 2: KEGG\_ADHERENS\_JUNCTION      
 Blue-Pink O' Gram in the Space of the Analyzed GeneSet

  

Fig 3: KEGG\_ADHERENS\_JUNCTION: Random ES distribution      
 Gene set null distribution of ES for **KEGG\_ADHERENS\_JUNCTION**
